# Computational Models of Articular Cartilage

**DOI:** 10.1155/2013/254507

**Published:** 2013-04-30

**Authors:** Rami K. Korhonen, Petro Julkunen, LePing Li, Corrinus C. van Donkelaar

**Affiliations:** ^1^Department of Applied Physics, University of Eastern Finland, P.O. Box 1627, 70211 Kuopio, Finland; ^2^Department of Clinical Neurophysiology, Kuopio University Hospital, P.O. Box 1777, 70211, Kuopio, Finland; ^3^Department of Mechanical and Manufacturing Engineering, University of Calgary, Calgary, AB, Canada T2N 1N4; ^4^Department of Biomedical Engineering, Eindhoven University of Technology, P.O. Box 513, 5600 MB Eindhoven, The Netherlands

In osteoarthritis, articular cartilage degenerates and eventually wears out, resulting in pain and disability. It is a major challenge in health care to prevent osteoarthritis or slow down the progression of the disease. The onset of osteoarthritis may result from an altered stress and strain state in cartilage, for example, due to an injury of ligament, cartilage, or meniscus. However, the disease progression is patient specific and can hardly be predicted. In order to assess the articular cartilage function and possible failure sites in joints, and to evaluate the onset and progression of osteoarthritis patient-specifically, computational models may become useful tools. Although there has been significant progress over the last years, these models need to be further developed before they may eventually become of direct clinical value. 

For clinical, patient-specific application of a computer model, a realistic description of joint geometry, joint kinematics, and tissue loading is ultimately required. Geometrical data can be obtained from imaging modalities, such as MRI, which become increasingly more detailed. Still, meshing of such information and running computational simulations of such meshes is challenging. Simulation analysis of joint kinematics is computationally expensive and requires knowledge of the kinematic constraints as a consequence of muscles and ligaments, including, for instance, pre stresses and attachment sites of ligaments. In many cases, 3D and/or kinematic simulations are still more feasible in combination with simpler material models for cartilage. More advanced material descriptions are required for a more detailed investigation of mechanical conditions inside cartilage. However, these are essential to move forward in our understanding of the relationship between mechanical loading, cartilage damage, and osteoarthritis. 

A wide variety of cartilage models have been developed and employed to evaluate the mechanical behavior at the cell, tissue, and joint level. They have been used to evaluate static and dynamic tissue behavior, to explore effects of mechanical and biochemical loading, and even to predict tissue adaptation at the long time scale. The expectation is that such models may be taken to the next level, where patient-specific characteristics are incorporated in 3D kinematic joint models. 

Given the composite structure of cartilage with the collagen fibril network, proteoglycan matrix, and fluid, this special issue has a focus on the development and application of fibril-reinforced models at different scales. A review of the fibril-reinforced tissue models and their application in the cell level is presented (P. Julkunen et al.). Practical considerations in modeling applications and model limitations are further discussed. A thorough review of recent advances in computational modeling of human knee joints is also presented (M. Kazemi et al.). Creation of knee joint models starting from imaging data (such as MRI) is summarized, clinical applications of the models are discussed, and examples are given for patient-specific evaluation of knee joint mechanics. Furthermore, influence of the altered depth-dependent properties of cartilage in osteoarthritis on fluid pressurization in a knee joint is demonstrated (Y. Dabiri and L. P. Li). Since generation of computational finite element models of knee joints is time-consuming and model simulation times can be relatively long, a semiautomated method is presented to study tibiofemoral contact stresses of adult subjects (D. D. Anderson et al.). 

Combination of experimental measurements and modeling is reviewed and model validation in cell, tissue, and joint level is discussed (P. Julkunen et al. and M. Kazemi et al.). Specifically, experimental results on the depth-dependent microstructural response of cartilage under directly loaded and unloaded regions are presented (A. Thambyah and N. Broom). Loading-related changes in chondron aspect ratios at different zones are also shown. These results can be used for the validation and development of computational models incorporating higher degrees of structural realism. Further, experimentally measured chondrocyte deformation behavior under mechanical loading of the tissue is presented for normal and osteoarthritic cartilage (P. Tanska et al.). Computational multiscale modeling is applied to explain differences in the cell behavior between the aforementioned groups. Finally, potential future directions in the application of multiscale models for the evaluation of cell responses and disease progression in knee joints are addressed (P. Julkunen et al.).

 Recent advances in the development of computational models of articular cartilage, as partially demonstrated in this special issue, and models of joints with realistic patient-specific material descriptions and joint kinematics have brought clinical application closer. More advanced adaptation models, likely combinations of models addressing different length scales, should ultimately lead to the prediction of altered tissue properties and wear, which strongly correlate to the development of osteoarthritis. Ultimately, computational modeling may become a clinical tool for identifying or optimizing patient-specific treatment strategies, such as rehabilitation and surgical interventions.

*Rami K. Korhonen* *Petro Julkunen* *LePing Li* *Corrinus C. van Donkelaar*

